# Reviewer acknowledgement 2015

**DOI:** 10.1186/s12938-016-0130-0

**Published:** 2016-02-24

**Authors:** Ken Foster, Fong-Chin Su

**Affiliations:** BioMedical Engineering OnLine, Department of Bioengineering, University of Pennsylvania, Philadelphia, PA USA; BioMedical Engineering OnLine, Department of Biomedical Engineering, National Cheng Kung University, Tainan, Taiwan

*BioMedical Engineering OnLine* would like to thank all reviewers who have contributed to the journal in 2015.

**Eduardo Abreu**

USA

**U Rajendra Acharya**

Singapore

**Anusha Achuthan**

Malaysia

**Mohd Yazed Ahmad**

Malaysia

**Hulusi Acikgoz**

Turkey

**Md Rezwanul Ahsan**

Malaysia

**M Eren Ahsen**

USA

**S Akram**

Pakistan

**Muhammad Usman Akram**

Pakistan

**Walid Al-Atabany**

Egypt

**S Alavi**

USA

**Prochazka Ales**

Czech Republic

**Ali Al-Haj**

Jordan

**David Alonso-Caneiro**

Australia

**Fernando Andreotti**

Germany

**Javier Mauricio Antelis**

Mexico

**Daisuke Anzai**

Japan

**Peter Aquilina**

Australia

**Adamantios Arampatzis**

Germany

**Carmen Paz Araujo**

Spain

**Muhammad Arif**

Saudi

**Thomas Armstrong**

USA

**Mustafa Atac**

Turkey

**David Attaway**

USA

**Hug Aubin**

Germany

**Helene Autefage**

UK

**Wajid Aziz**

Pakistan

**Zahra Bagheri**

Canada

**Merete Bakke**

Denmark

**Rupak Banerjee**

USA

**Peña Baquedano**

Spain

**Jayme Barbedo**

Brazil

**Nick Barnes**

Australia

**Philipp Berg**

Germany

**Leopold Berger**

Austria

**Pinaki Bhattacharya**

Belgium

**Gili Bisker**

USA

**Michal Borecki**

Poland

**Emrah Bostan**

Switzerland

**Richard Bostelmann**

Germany

**Patrick Boyle**

USA

**Annelies Bronckaers**

Belgium

**Francesca Buganè**

Italy

**Vladimír Bureš**

Czech Republic

**Martin Burger**

Germany

**Kevin Burrage**

Australia

**Kevin Burrage**

UK

**Martin Burtscher**

Austria

**L Canedo-Dorantes**

Mexico

**C R Caridade**

Portugal

**Maria Claudia Castro**

Brazil

**Piergiorgio Cerello**

Italy

**Nai-Jen Chang Chang**

Taiwan

**Chih-Wei Chang**

Taiwan

**Thanatip Chankong**

USA

**Pen-hsiu Chao**

Taiwan

**Geoff Chase**

New Zealand

**Sichang Chen**

China

**Ying Chen**

China

**Yiping Chen**

China

**Po-Tsun Chen**

Taiwan

**Yuan-Fu Chen**

Taiwan

**Kuo-Sheng Cheng**

Taiwan

**Renee Chin**

Malaysia

**Wookjin Choi**

USA

**Cheng-Ying Chou**

Taiwan

**Kian Jon Chua**

Singapore

**Han-Sheng Chuang**

Taiwan

**Edward Ciaccio**

USA

**Ross Clark**

Australia

**Gari Clifford**

UK

**Matthijs Cluitmans**

Netherlands

**P Coan**

Germany

**Claudio Cobelli**

Italy

**Bryan Cole**

USA

**Gillian Crofts**

UK

**Krzysztof Cyran**

Poland

**Houde Dai**

China

**L’uboš Danišovič**

Slovakia

**Mayank Dave**

India

**Kathryn Davis**

USA

**Antonio De Carvalho Filho**

Brazil

**G de Haan**

Netherlands

**Maria De Miguel**

Spain

**Giorgio De Nunzio**

Italy

**Jesse Dean**

USA

**Stefan Debener**

Germany

**Richard Debski**

USA

**Sudhir Deosarkar**

USA

**Thomas Desaive**

Belgium

**Marco Di Rienzo**

Italy

**Mary Díaz**

Venezuela

**Irraivan Elamvazuthi**

Malaysia

**Moe Elgendi**

Canada

**Marwan El-Rich**

Canada

**Gordon A Ewy**

USA

**Jiabing Fan**

USA

**Rosse Mary Falcón-Antenucci**

Brazil

**C G Farmer**

USA

**Willem Fennis**

Netherlands

**Nenad Filipovic**

Serbia

**Kenneth Foster**

USA

**Frederique Frouin**

France

**Yang-Chieh Fu**

USA

**Humberto Gamba**

Brazil

**Johan Gaverth**

Sweden

**Amit Gefen**

Israel

**Piergiorgio Gentile**

UK

**Brendan Geraghty**

UK

**Frank Gijsen**

Netherlands

**Maria Helena Gil**

Portugal

**Rizzo Giovanna**

Italy

**Larry R Glazerman**

USA

**Maria Jose Gomez-Benito**

Spain

**He Gong**

China

**Haiyan Gong**

USA

**Carla Goy**

Argentina

**Sibylle Grad**

Switzerland

**Boris Gramatikov**

USA

**Eyal Gruntman**

USA

**Lan-Yuen Guo**

Taiwan

**Viatcheslav Gurev**

USA

**Neda Haj-Hosseini**

Sweden

**Jae Yong Han**

Germany

**Md Mehedi Hasan**

Canada

**Kamran Hassani**

Iran

**Jens Haueisen**

Germany

**Jan Havlik**

Czech Republic

**Bin He**

USA

**Li He**

USA

**Roy Hessels**

Netherlands

**Monica Hinds**

USA

**Lynette Hodges**

New Zealand

**Keum-Shik Hong**

South Korea

**Eun-Kyoung Hong**

USA

**Chih-Kun Hsiao**

Taiwan

**Hao-Ming Hsiao**

Taiwan

**Tsung-Hsun Hsieh**

Taiwan

**Yi-Zeng Hsieh**

Taiwan

**Wei-Chun Hsu**

Taiwan

**Wei-Li Hsu**

Taiwan

**Jinlian Hu**

Hong Kong

**Jin-Jia Hu**

Taiwan

**Sijung Hu**

UK

**Meghan Huber**

USA

**Ing-Shiou Hwang**

Taiwan

**Tadashi Ihara**

Japan

**Trienke Ijmker**

Netherlands

**Jason Inzana**

Switzerland

**Md Saiful Islam**

Saudi Arabia

**Clara Ionescu**

Belgium

**Cinthia Itiki**

Brazil

**Richard Jaspers**

Netherlands

**Joonsoo Jeong**

South Korea

**Hueihan Jhuang**

Taiwan

**Ming Ju**

Taiwan

**Shi Jun**

China

**Sung Jun**

South Korea

**Foad Kabinejadian**

Singapore

**Muammar Kabir**

USA

**Yasser Kadah**

Egypt

**Lyes Kadem**

Canada

**Sei-ichiro Kamata**

Japan

**Alexey Kamenskiy**

USA

**Bilge Karacali**

Turkey

**Tahir Karaman**

Turkey

**Dan Stieper Karbing**

Denmark

**Igor Karsaj**

Croatia

**Jubin Kashef**

Germany

**N Kawai**

Japan

**Hyung Joo Kim**

South Korea

**Jungsuk Kim**

South Korea

**Young Eun Kim**

South Korea

**Sunghan Kim**

USA

**Sungwan Kim**

USA

**Yo Kishimoto**

Japan

**Zelma Kiss**

Canada

**Seokbum Ko**

Canada

**Kranthi Kolli**

USA

**Robert Koprowski**

Poland

**Kenichiro Koshiyama**

Japan

**Algimantas Krisciukaitis**

Lithuania

**Inger Kuhn**

Sweden

**Laurel Kuxhaus**

USA

**Oh-Yun Kwon**

South Korea

**Douglas Lake**

USA

**Michele Lanza**

Italy

**Heow Pueh Lee**

Singapore

**Jinseok Lee**

South Korea

**Pei-Yun Lee**

Taiwan

**Tzer-Min Lee**

Taiwan

**Su-Ya Lee**

Taiwan

**Haifeng Li**

China

**Jingfeng Li**

China

**Junbo Li**

China

**Qingli Li**

China

**Tong Li**

China

**John Li**

USA

**F Y Liang**

China

**Cheng-Li Lin**

Taiwan

**Hwai-Ting Lin**

Taiwan

**Chien-Ju Lin**

Taiwan

**Cheng-Feng Lin**

Taiwan

**Chun-Li Lin**

Taiwan

**Sonia Lippke**

Germany

**Guoqiang Liu**

China

**Jie Liu**

China

**Wenyong Liu**

China

**X M Liu**

China

**Alberto Llera**

Netherlands

**Nicolas Lomenie**

France

**Jianwen Luo**

China

**Leonidas Lymberopoulos**

Greece

**Warren Macdonald**

UK

**Malcolm Macleod**

USA

**D Major**

Austria

**João Mano**

Portugal

**Francois Marchal**

France

**Kendal Marriott**

Canada

**Joan Marti**

Spain

**Ramon Martinez**

USA

**Raul Alcaraz Martinez**

Spain

**Takeshi Matsumura**

Japan

**Christopher Mayer**

Austria

**Pedro Melo**

Brazil

**Fabrice Meriaudeau**

France

**Mena Mesiha**

USA

**Fen Miao**

China

**Francesco Migliavacca**

Italy

**Shogo Minagi**

Japan

**Hsieh Ming-Fa**

Taiwan

**Hiroshi Mitoma**

Japan

**Hideki Mizu-Uchi**

Japan

**Fernando Moraga**

Bangladesh

**Rosario Morello**

Italy

**Matteo Moretti**

Italy

**Dylan Morrissey**

UK

**Yosry (Yos) Morsi**

Australia

**Simon Moulton**

Australia

**Tetsuro Muraoka**

Japan

**Krzysztof Murawski**

Poland

**F A D Nascimento**

Brazil

**H Naseri**

Iran

**Mohammadreza Nasiriavanaki**

USA

**Raghu N Natarajan**

USA

**Eduardo Naves**

Brazil

**Kianoush Nazarpour**

UK

**Nathan Neckel**

USA

**Dong Ni**

China

**Jennifer Nichols**

USA

**David Nickerson**

New Zealand

**Wioletta Nowak**

Poland

**Jim O’Doherty**

UK

**Ikenna Odinaka**

USA

**Rei Ogawa**

Japan

**Nick Oliver**

UK

**David Pablo Piñero Llorens**

Spain

**José Blas Pagador**

Spain

**Ramaswamy Palaniappan**

UK

**Luca Palmerini**

Italy

**Gustavo Pamplona**

Brazil

**H C Pan**

Taiwan

**Shing-Tai Pan**

Taiwan

**Michail Papafaklis**

Greece

**Jinhyoung Park**

USA

**Jeremy Patterson**

USA

**Robert Patterson**

USA

**Gürel Pekkan**

Turkey

**Rafael Pena-Miller**

Mexico

**Antonio Pérez-González**

Spain

**Thilo Pfau**

UK

**Lacomme Philippe**

France

**Angkoon Phinyomark**

Canada

**Peter Pivonka**

Australia

**Peter Plassmann**

UK

**Christopher Pretty**

New Zealand

**Ales Prochazka**

Czech Republic

**Rita Pucci**

Italy

**Shouliang Qi**

China

**Xin Qi**

USA

**Aike Qiao**

China

**Jing Qin**

China

**Muhannad Quwaider**

Jordan

**Ahmed Radwan**

USA

**Benyamin Rahmani**

UK

**Jaime Ramírez-Vick**

USA

**Marjan Razani**

Canada

**Stephen Redmond**

Australia

**Marwan Refaat**

USA

**Francisco Renero**

Mexico

**Claus-Peter Richter**

USA

**Carina Riediger**

Germany

**Alicia Rodríguez-Barbero**

Spain

**Verneri Ruonala**

Finland

**William Rymer**

USA

**Yoshifumi Saijo**

Japan

**Takao Saito**

Japan

**Solomon Samuel**

USA

**Saeid Sanei**

UK

**Cristina Santos**

Portugal

**M Iqbal Saripan**

Malaysia

**Denis Scaini**

Italy

**Lukas Schaupp**

Austria

**Reinhold Scherer**

USA

**Holger Schmidt**

Germany

**T Seo**

North Korea

**Maxime Sermesant**

France

**Sharmishtaa Seshamani**

USA

**Salvatore Sessa**

Japan

**Eva Sevick**

USA

**M Shahbakhti**

Iran

**Amir Akramin Shafie**

Malaysia

**Guofa Shou**

USA

**Alberto Signoroni**

Italy

**Pekka Siirtola**

Finland

**Andrzej Skowron**

Poland

**Roland Smith**

USA

**Manuchehr Soleimani**

UK

**Ahmed Soliman**

Egypt

**L C Sousa**

Portugal

**M Spuler**

Germany

**Carol Stanciu**

Romania

**R Steele**

USA

**Joanna Stephen**

UK

**Ulrich Stock**

Germany

**Radovan Stojanovic**

Montenegro

**Peter Stubbs**

Denmark

**Kuo-Chih Su**

Taiwan

**P V Sudeep**

India

**Zhonghua Sun**

Australia

**Cheuk-Kwan Sun**

Taiwan

**Tamara Supuk**

Croatia

**Hadi Tadayyon**

Canada

**Ta-Wei Tai**

Taiwan

**Jeffrey Tan**

Australia

**Dalin Tang**

USA

**Junhua Tang**

USA

**Erdal Tasci**

Turkey

**David Thompson**

USA

**Lan Tian**

China

**Thorsten Tjardes**

Germany

**Adriano Tort**

Brazil

**Maria Trocan**

France

**Kun-Ling Tsai**

Taiwan

**Yi-Ju Tsai**

Taiwan

**Yung-Shen Tsai**

Taiwan

**Yi-Jung Tsai**

Taiwan

**Tsung-Yuan Tsai**

USA

**Stavros Tsantis**

Greece

**Alexander Tsouknidas**

Greece

**Matthew Urban**

USA

**Álvaro Valencia**

Chile

**Moreno Valenzuela**

Mexico

**J J van Rheede**

UK

**Saeed Vaseghi**

UK

**Alberto Vazquez**

USA

**Jussi Virkkala**

Finland

**Marios Vlachos**

Greece

**Aleksandra Vuckovic**

UK

**Martha Walker**

USA

**J J Wang**

China

**Weidong Wang**

China

**Yan Wang**

China

**Defeng Wang**

Hong Kong

**Qian Wang**

USA

**Qingzhu Wang**

USA

**Ashley Anne Weaver**

USA

**Y X Wei**

China

**George K C Wong**

Hong Kong

**Jerzy Wtorek**

Poland

**Chengtie Wu**

China

**Jianhuang Wu**

China

**Zhong Wu**

China

**Josh Wu**

Taiwan

**Hong-Bo Xie**

Australia

**Wenwei Xu**

USA

**Fraid Yaghouby**

USA

**Bin Yan**

China

**Zhi Yang**

China

**Saami Yazdani**

USA

**Ming-Long Yeh**

Taiwan

**Joon Hock Yeo**

Singapore

**Erwei Yin**

China

**Mitsuru Yoneyama**

Japan

**Jia-Yuan You**

Taiwan

**Wonsang You**

USA

**Azizeh-Mitra Yousefi**

USA

**Romano Zannoli**

Italy

**Sebastian Zaunseder**

Germany

**Hongwu Zeng**

China

**Guanglei Zhang**

China

**Qi Zhang**

China

**Yi Zhang**

China

**Kaiyin Zhang**

USA

**Shijia Zhang**

USA

**Zhilin Zhang**

USA

**Linping Zhao**

USA

**G Y Zheng**

Switzerland

**Changchun Zhou**

China

**Zhenyu Zhou**

China

**Zhongxing Zhou**

China

**Danhua Zhu**

USA

**Marvin Ziskin**

USA

